# The anti-bacterial iron-restriction defence mechanisms of egg white; the potential role of three lipocalin-like proteins in resistance against *Salmonella*

**DOI:** 10.1007/s10534-019-00180-w

**Published:** 2019-02-27

**Authors:** Louis Alex Julien, Florence Baron, Sylvie Bonnassie, Françoise Nau, Catherine Guérin, Sophie Jan, Simon Colin Andrews

**Affiliations:** 1 School of Biological Sciences, University of Reading, Reading, RG6 6UA UK; 2 UMR1253 Sciences et Technologie du Lait et de l’œuf, Agrocampus-Ouest/INRA, 35042 Rennes, France; 3 Université de Rennes I, Rennes, France

**Keywords:** *Salmonella* Enteritidis, Salmochelin, Enterobactin, Ex-FABP, Cal-γ, Alpha-1-ovoglycoprotein

## Abstract

*Salmonella* enterica serovar Enteritidis (*S*E) is the most frequently-detected *Salmonella* in foodborne outbreaks in the European Union. Among such outbreaks, egg and egg products were identified as the most common vehicles of infection. Possibly, the major antibacterial property of egg white is iron restriction, which results from the presence of the iron-binding protein, ovotransferrin. To circumvent iron restriction, *S*E synthesise catecholate siderophores (i.e. enterobactin and salmochelin) that can chelate iron from host iron-binding proteins. Here, we highlight the role of lipocalin-like proteins found in egg white that could enhance egg-white iron restriction through sequestration of certain siderophores, including enterobactin. Indeed, it is now apparent that the egg-white lipocalin, Ex-FABP, can inhibit bacterial growth via its siderophore-binding capacity in vitro. However, it remains unclear whether Ex-FABP performs such a function in egg white or during bird infection. Regarding the two other lipocalins of egg white (Cal-γ and α-1-glycoprotein), there is currently no evidence to indicate that they sequester siderophores.

## Survival of *Salmonella* within egg white and the role of iron restriction

### The powerful antibacterial defence mechanisms of egg white

In the EU, *Salmonella enterica* is the bacterium most frequently (93%) detected in egg white, with *S. enterica* serovar Enteriditis (*S*E) being the major (67%) strain associated with outbreaks caused by eggs and egg products (EFSA 2014). Thus, *S*E appears to be very well suited to infection of, and survival within, eggs (Clavijo et al. 2006; Vylder et al. 2013; Gantois et al. 2008). Egg white is noted for its strong antimicrobial activity which indicates that *S*E has powerful egg-white resistance mechanisms. Indeed, the various antimicrobial activities exhibited by egg white can be considered to present a unique set of challenges for bacterial invaders. These include physico-chemical factors, in particular high pH (inhibiting growth; Sharp and Whitaker 1927) and high viscosity (limiting motility; Schneider and Doetsch 1974; Yadav and Vadehra 1977); in addition, high osmolarity (causing osmotic stress) has been suggested (Clavijo et al. 2006). The pH of egg white shifts from ~ 7.6 (upon oviposition) to 9.3 (a few days later) as a result of CO_2_ release (Sharp and Powell 1931). The viscosity of egg white (with a shear rate of 400 s^−1^: 5 mPa.s^−1^ at 20 °C; Lang and Rha 1982) is mainly caused by the presence of ovomucin, a glycoprotein contributing 3.5% w/w of the total egg albumin protein (egg white has a total protein content of ~ 10% w/w; Kovacs-Nolan et al. 2005).

In addition to these physico-chemical factors, egg white possesses an array of proteins that provide further defence against pathogens [see review by Baron et al. (2016) for more detail], notably:

- ovotransferrin (oTf), involved in iron deprivation (Garibaldi 1970) and bacterial membrane damage (Aguilera et al. 2003);
- lysozyme (Derde et al. 2013) and defensins (Hervé-Grépinet et al. 2010; Gong et al. 2010), that would be expected to disrupt bacterial membrane integrity;
- ovalbumin X, a heparin-binding protein exhibiting antimicrobial activity (Réhault-Godbert et al. 2013);
- ovostatin (Nagase et al. 1983) and cystatin (Wesierska et al. 2005), presumed to inhibit exogenous proteases; and
- avidin (Banks et al. 1986), a biotin sequestration protein.

### The major role of iron restriction and ovotransferrin in egg-white defence

It is generally accepted that the major factor limiting bacterial growth in egg white is iron restriction. This results from the presence of oTf, a powerful iron-binding protein (Garibaldi 1970; Lock and Board 1992; Baron et al. 1997). The iron restriction of egg white was first discovered by Schade and Caroline (1944) who found that exposure to egg white inhibits the growth of *Shigella dysenteriae.* Among 31 growth factors added to egg white, only iron overcame the observed egg white-imposed growth inhibition. Two years later, Alderton et al. (1946) identified the egg-white factor responsible as ‘conalbumin’, which is now known to be a member of the transferrin family and is more commonly referred as oTf. Since these early studies, subsequent work has confirmed the role of oTf as an egg-white iron-restriction agent preventing growth of a range of microbial species, including *Salmonella* (Schade and Caroline 1944; Valenti et al. 1983, 1985; Ibrahim 1997; Baron et al. 1997, 2000). Indeed, iron-acquisition mutants of *S*E display decreased survival and/or growth in egg white (Kang et al. 2006). Such studies confirm the antibacterial role of iron restriction in egg white. A recent global transcriptomic study (Baron et al. 2017) revealed a major iron-starvation response of *S*E upon exposure to egg white which was caused by relief of Fur- (the global transcriptional regulator of iron-dependent gene expression; Rabsch et al. 2003) mediated repression. Likewise, a quantitative proteomic analysis (isobaric tags for relative and absolute quantitation; iTRAQ) showed that iron-acquisition-system-related proteins are induced by egg white (Qin et al. 2019). These findings confirm that *S*E suffers from iron limitation in egg white. The low iron availability in egg white exerts a strong bacteriostatic influence (Bullen et al. 1978; Baron et al. 1997) because iron is essential for growth of nearly all organisms, including bacteria (Andrews et al. 2003). In many ways, the antibacterial iron-restriction strategy of egg white is comparable to the iron-dependent ‘nutritional immunity’ defence mechanisms observed in mammals, where serum transferrin maintains concentrations of extracellular free iron at levels (10^−18^ M) well below those that support bacterial growth (Bullen et al. 2005).

OTf is believed to be the critical iron-restriction component in egg white. Like other members of the transferrin family, its structure consists of two ‘lobes’, each with a strong affinity for a single Fe^3+^ ion (apparent binding constant of around 10^32^ M^-1^, with of 1.5 × 10^18^ M^-1^ and 1.5 × 10^14^ M^-1^ for the C- and N-terminal lobes, respectively, at pH 7.5; Guha-Thakurta et al. 2003; Chart 1993; Schneider et al. 1984). The iron-restriction-based bacteriostatic activity of oTf is enhanced by bicarbonate (which is likely related to the apparent dependence of metal binding on the presence of a suitable anion; Valenti et al. 1983) and high pH (Valenti et al. 1981; Antonini et al. 1977; Lin et al. 1994). Interestingly, egg white contains levels of iron (~ 0.1 mg iron per 100 g which is equivalent to ~ 18 µM; USDA 2010; Nys and Sauveur 2004) that would normally be sufficient for bacterial growth. However, oTf is present in such high abundance in egg white (170 µM; 13% of total protein content, second most abundant egg white protein after ovalbumin; Sauveur 1988) that oTf iron-binding capacity exceeds iron availability by 17-fold. Since egg white is aerobic and has a high pH (after laying), iron in egg white would be expected to be largely in the ferric state (the form bound by oTf) so it can be assumed that virtually all iron in egg white is bound to oTf such that very little is freely available (Sauveur 1988). However, some bacteria are more susceptible to growth inhibition by oTf than others. Indeed, in vitro studies showed that the most sensitive species are *Pseudomonas* and *Escherichia coli*, and the most resistant are *Staphylococcus aureus, Proteus* and *Klebsiella* (Valenti et al. 1983). Unsurprisingly, the effects of oTf can be relieved by iron-mobilising agents (e.g. citrate) (Valenti et al. 1983). OTf also appears to possess additional antibacterial activities since its effects are diminished when separated from direct contact with bacteria through location in a dialysis bag or immobilisation on beads (Valenti et al. 1985). Indeed, it has been shown that oTf can partially penetrate and permeabilise bacterial membranes, acting as an uncoupling agent (Aguilera et al. 2003). This activity is likely related to the presence of a Cys-rich antibacterial-peptide-like motif located on the surface of the oTf molecule which confers the ability to kill Gram-negative bacteria (Ibrahim et al. 1998, 2000).

## Iron acquisition by *Salmonella*

### The role of the two siderophores of *Salmonella* in iron uptake and pathogenicity

The most important mechanism used by bacteria to circumvent iron restriction involves the synthesis of siderophores that bind exogenous ferric iron with high affinity and specificity, and enable acquisition of iron from host sources (Andrews et al. 2003). The siderophores employed by *Salmonella* are catecholates called enterobactin (or enterochelin) and salmochelin. Enterobactin was first identified in *E. coli* (O’Brien and Gibson 1970) and *Salmonella* Typhimurium (*S*T) (Pollack and Neilands 1970). Although enterobactin synthesis was shown to be required for survival of *S*T in low-iron in vitro environments (Pollack et al. 1970), its role in pathogenesis is limited for reasons that were, initially, unclear (Benjamin et al. 1985; Rabsch et al. 2003). Salmochelin (which is closely similar to enterobactin) was not identified until more than three decades after enterobactin when it was found to be a product of pathogenic enterobacteria, such as *Salmonella* (Hantke et al. 2003). It was designated ‘salmochelin’ as it appeared at first to be a characteristic of *Salmonella* strains. However, salmochelins have now been reported in avian pathogenic *E. coli* (APEC), uropathogenic *E. coli* (UPEC), *S*T and *Klebsiella pneumoniae* where they contribute to virulence (Caza et al. 2008; Gao et al. 2012; Crouch et al. 2008; Bachman et al. 2012). It should be noted that the ferrous-iron transport systems of *Salmonella* (FeoABC and SitABCD) can also contribute to pathogenicity and/or gut colonisation (see review by Carpenter and Payne 2014).

### Enterobactin: a powerful siderophore, but with limited effect in vivo

**Fig. 1:**
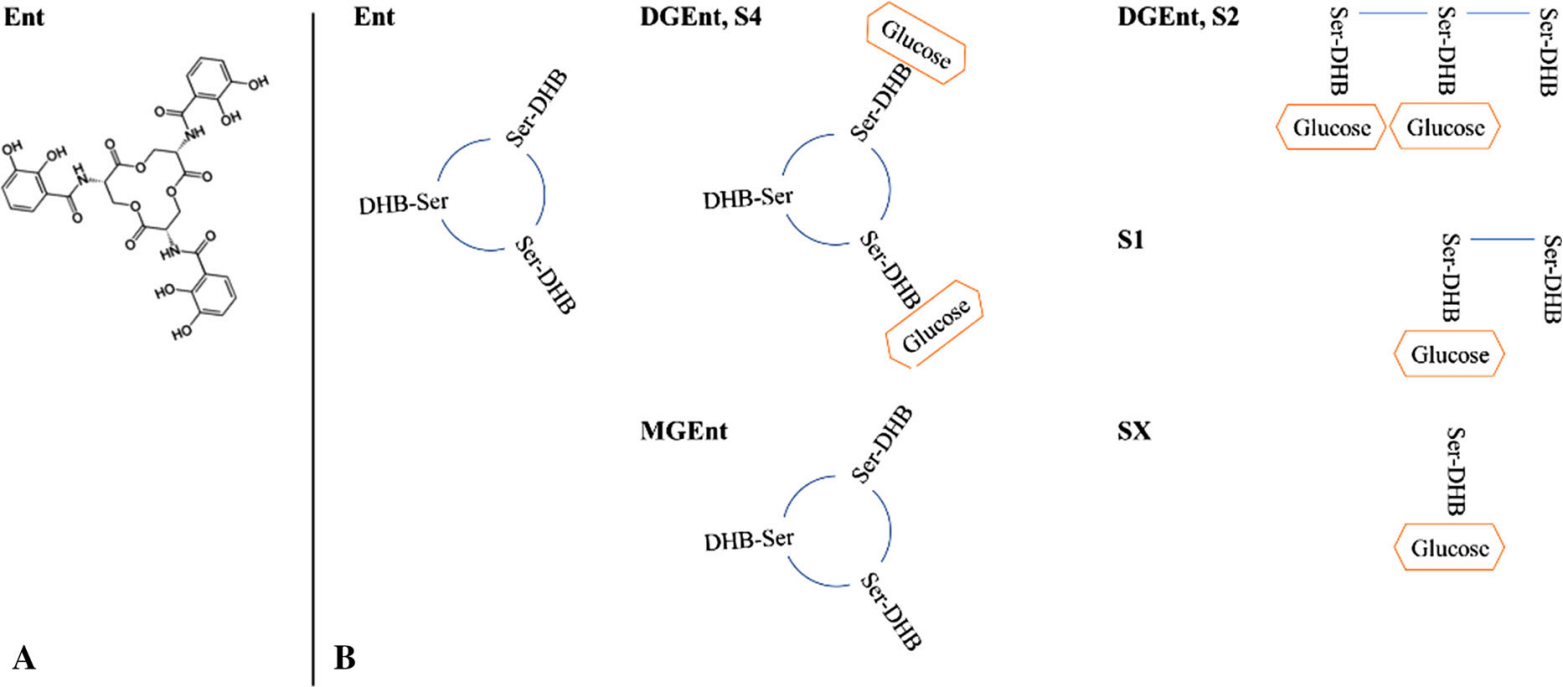
Enterobactin (Ent), Salmochelin (S4) and related structures. **a** Structure of enterobactin. **b** Schematic representation of Ent, S4 and its derivatives. Salmochelin S4 and S2 are both di-glucosylated forms of enterobactin (DGEnt) but the latter is linear. MGEnt is a 2,3-dihydroxybenzoyl serine macrotrilactone that is glucosylated only once. The salmochelin degradation products, S1 and SX are the mono-glucosylated dimer and monomer, respectively. *Ser* serine, *DHB* dihydroxybenzoate

**Fig. 2:**
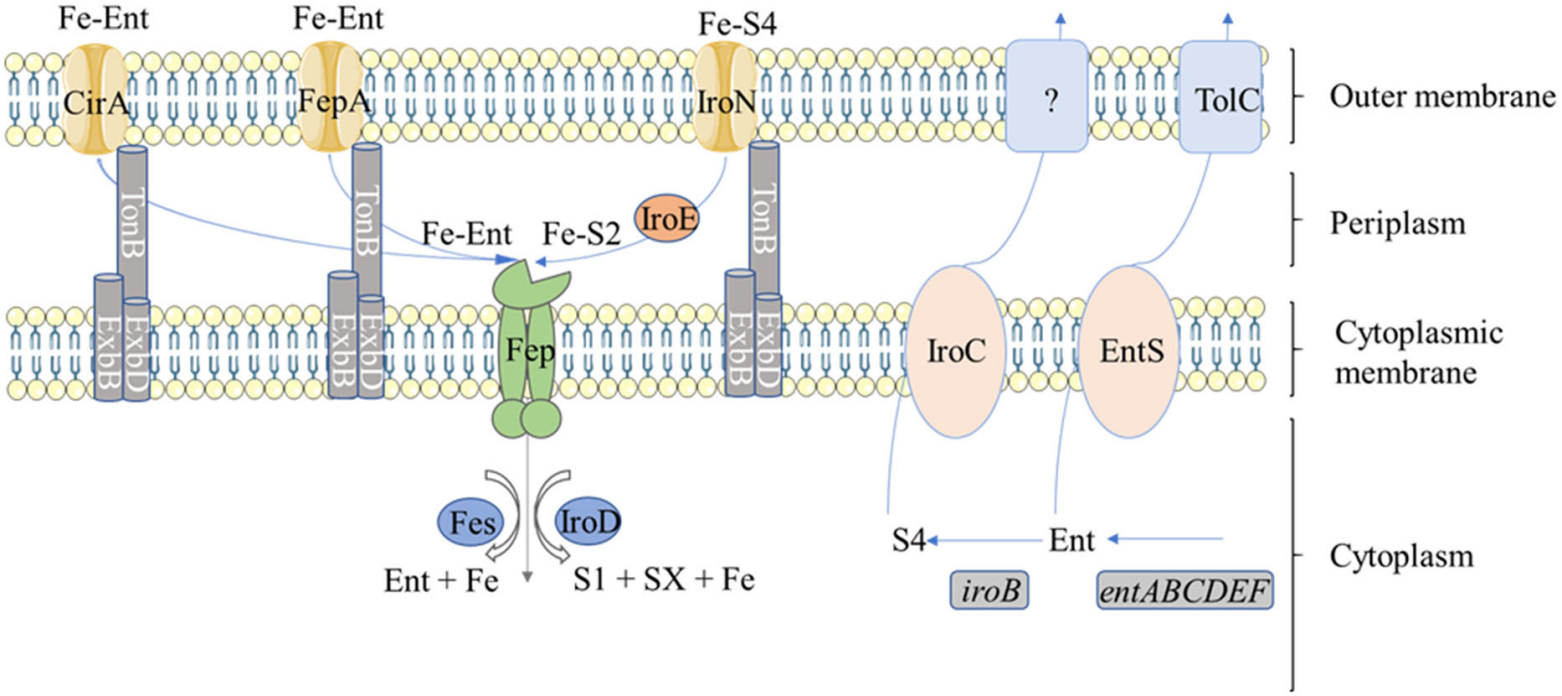
Summary of synthesis, export, import and utilisation, of enterobactin and salmochelin. Enterobactin (synthesised by EntABCDEF) is mono or di-glucosylated by IroB. Enterobactin is exported from the cytoplasm by EntS whereas salmochelin is exported by IroC. TolC is then involved in enterobactin efflux across the outer membrane. Once complexed with ferric iron, ferri-salmochelin is taken up across the outer membrane via IroN and is then linearized to the S2 form by the periplasm IroE esterase, whereas ferri-enterobactin is taken up via CirA, or FepA. The Fe-S2 and Fe-Ent are then transported into the cytoplasm via the FepBCDG ATP-binding cassette transporter, and are then esterified by the IroD and Fes esterases, respectively, which is presumed to facilitate iron release. The resulting salmochelin degradation products, S1 and SX, are exported from the cytoplasm into the medium. It is important to note that the Iro transport components can also assist with enterobactin utilisation

Enterobactin is a serine macrotrilactone (Fig. 1) that has a far higher affinity for iron than oTf (formation constants of 10^52^ and 10^32^ M^-1^, respectively), which allows siderophore-producing bacteria to use oTf as a source of iron (Chart 1993). Although the metabolism of enterobactin is best studied in *E. coli*, *Salmonella* possesses a highly similar set of enterobactin-related genes which are assumed to play similar roles. The enterobactin precursor, 2,3-dihydroxybenzoate (DHB), is synthesized from chorismate by enzymes encoded by the *entC*, *entB* and *entA* genes. In a second step, DHB and serine are combined, polymerized and cyclized to form enterobactin by enzymes encoded by the *entE*, *entB* and *entF* genes (Gehring et al. 1998). EntS is required for enterobactin export through the cytosolic membrane (Furrer et al. 2002) whereas TolC is involved in enterobactin efflux across the outer membrane (Bleuel et al. 2005) (Fig. 2). Once complexed with ferric iron, uptake of ferric-enterobactin into the periplasm is mediated by the iron-regulated outer-membrane proteins, FepA and Cir (and IroN in *Salmonella*) (Rabsch et al. 1999, 2003); the energy-transducing TonB-ExbBD complex is also required for this step (Skare et al. 1993; Fig. 2). Ferric-enterobactin is then imported into the cytoplasm by the ATP-binding cassette transporter, FepBDGC (Langman et al. 1972; Chenault and Earhart 1992). Finally, the imported ferric-enterobactin complex is processed by the Fes esterase which cleaves the cyclic ring of the siderophore, lowering affinity for the bound iron which enables dissociation (O’Brien et al. 1971).

Despite its high affinity for iron, enterobactin is not as effective as other siderophores in vivo (Konopka et al. 1982; Montgomerie et al. 1984), and this poor performance appears to be related to its rapid clearance from the serum (Konopka and Neilands 1984). An unknown factor in serum was found to impede transfer of iron from transferrin to enterobactin, and from ^55^Fe-enterobactin to *E. coli* (Konopka and Neilands 1984). However, serum has little impact on iron chelation by the aerobactin siderophore (Konopka and Neilands 1984). Aerobactin was also shown to provide a significant selective advantage for *E. coli* growth in vitro (Williams and Carbonetti 1986), and in a cutaneous infection model (Demir and Kaleli 2004), even though its affinity for iron is weaker than that of enterobactin (formation constants of 10^23^ and 10^52^ M^-1^, respectively; Neilands, 1981). Similar findings were found for *S. enterica*, as enterobactin is not a virulence factor for *S*T or *S*E in mouse and chicken infection models (Benjamin et al. 1985; Rabsch et al. 2003). Later, the serum factor responsible for limiting the action of enterobactin was identified as an acute-phase protein, called LCN2 (lipocalin 2 or neutrophil gelatinase-associated lipocalin) (Goetz et al. 2002), that previously had an unclear specific purpose. LCN2 was subsequently found to be induced and secreted in response to activation of Toll-like innate immune receptors (Flo et al. 2004), bind to enterobactin (Goetz et al. 2002) and inhibit enterobactin activity partly through rapid clearance from the serum (Devireddy et al. 2005), and thus shown to function as a ‘siderocalin’ (siderophore-binding lipocalin).

### Salmochelin: a glucosylated siderophore, promoting Salmonella pathogenicity through LCN2 evasion

Salmochelin S4 is a diglucosyl-C enterobactin (Fig. 1). The affinity of salmochelin for Fe^3+^ is not reported (Valdebenito et al. 2006; Watts et al. 2012), however, it is assumed that glucosylation does not significantly impact Fe^3+^ ligation or affinity (Luo et al. 2006). The genetic locus responsible for this glucosylation of enterobactin in *Salmonella* is the *iro*-gene cluster (or ‘*iroA* locus’) (Hantke et al. 2003). This locus consists of two convergent transcription units: *iroBCDE* and *iroN* (Bäumler et al. 1998). Salmochelin synthesis involves the di-glucosylation of enterobactin into S4 in a step catalysed by the glucosyltransferase, IroB (Bister et al. 2004); the resulting salmochelin is then exported across the cytosolic membrane by IroC (Crouch et al. 2008; Fig. 2). Once complexed with ferric iron, ferric-salmochelin is taken up across the outer membrane via IroN (Hantke et al. 2003) and is subsequently linearized to the S2 form by the periplasm IroE esterase (Lin et al. 2005; Zhu et al. 2005). The S2 form is then transported into the cytoplasm via FepBCDG (which also imports enterobactin) (Crouch et al. 2008). The imported S2 salmochelin is further esterified by IroD into monomeric and/or dimeric forms, which is presumed to facilitate iron release (Lin et al. 2005; Zhu et al. 2005). The resulting degradation products, S1 and SX (mono-glucosylated dimer and monomer, respectively), are exported from the cytoplasm into the medium where they potentially contribute to iron acquisition (Lin et al. 2005; Zhu et al. 2005). Initially, the reason for the glucosylation of enterobactin (generating salmochelin) was unclear. Subsequently, it was discovered that although LCN2 has high affinity for enterobactin (K_d_ of 0.41 ± 0.11 nM) and its derivatives/precursors (DHB, K_d_ of 7.9 ± 1.8 nM), as well as other catecholate-type ferric siderophores (e.g. parabactin, cepabactin and carboxymycobactins; Goetz et al. 2002; Holmes et al. 2005), it does not effectively bind to salmochelin S4 (Fischbach et al. 2006; Valdebenito et al. 2007). Furthermore, the LCN2 receptor, 24p3, was shown to mediate the import of ferri-siderophore-bound LCN2 into mammalian cells, removing both the iron and enterobactin from circulation (Devireddy et al. 2005). Thus, glucosylation of enterobactin is considered to be a strategy employed by pathogens to prevent siderophore sequestration and removal from circulation by LCN2.

LNC2 was originally identified as a component of neutrophil granules but is also expressed in epithelial cells in response to inflammatory signals (Kjeldsen et al. 1993). Nielsen et al. (1996) revealed that LCN2 might bind lipophilic inflammatory mediators like platelet-activating factor, leukotriene B4 and lipopolysaccharide. This led to the initial suggestion that LCN2 acts as immune-modulatory factor through transport of lipophilic molecules to inflammation sites (Goetz et al. 2000). As eluded to above, a clearer purpose for LCN2 became apparent when the protein was produced heterologously in *E. coli* and was isolated bound, surprisingly, to a red chromophore, which was subsequently identified as enterobactin (Goetz et al. 2002). This finding led to further studies demonstrating a role for LCN2 in host–pathogen interactions (Bachman et al. 2012; Fischbach et al. 2006; Flo et al. 2004). These further studies showed that the *iro*-gene cluster confers resistance to the growth inhibitory effects of LCN2 in vitro and that mice rapidly succumb to infection by *E. coli* H9049 harbouring the *iro*-gene cluster, but not its *iro*-free counterpart (Fischbach et al. 2006). Other studies showed that salmochelin contributes to virulence of both avian pathogenic and uropathogenic *E. coli* (APEC and UPEC) through its iron-binding activity (Gao et al. 2012). Indeed, salmochelin-defective mutants of APEC E058 and UPEC U17 showed significantly decreased pathogenicity compared to the wild-type strains in a chicken infection model (Gao et al. 2012). Likewise, the efficient glucosylation (IroB), transport (IroC and IroN) and processing (IroD and IroE) of salmochelins were shown to be required for APEC virulence (Caza et al. 2008). The role of glucosylation in *S. enterica* pathogenicity was further illustrated by the observation that the *iro* locus confers a competitive advantage to *S*T in colonizing the inflamed intestine of wild-type, but not of LCN2-deficient, mice (Raffatellu et al. 2009). It should be noted that the glucosylation and linearisation of enterobactin was suggested to enhance the activity of salmochelin through increasing its hydrophilic nature, which might be advantageous for iron scavenging in a membrane-rich microenvironment (Luo et al. 2006).

## Egg-white ‘lipocalins’: role in enhancing iron restriction through sequestration of bacterial siderophores?

### Evidence for the presence of lipocalin-like proteins in egg white

LCN2 belongs to the ‘lipocalin superfamily’ which includes a variety of proteins involved in transport of hydrophobic ligands, such as purpurin, retinol-binding protein, α-1-glycoprotein, apolipoprotein, probasin, α-1-microglobulin and prostaglandin D synthetase. Although the family members display low overall sequence identity (Greene et al. 2003), lipocalins share a common three-dimensional structure characterised by an eight-stranded β-barrel (with a small C-terminal helix) that forms a chalice, at the bottom of which the hydrophobic ligand is bound (Françoise 1994). Due to their diversity, lipocalin-like proteins have various functions e.g. in immune response, pheromone transport, biological prostaglandin synthesis, retinoid binding and cancer cell interactions (Flower 1996). Lipocalins can be divided into two major subfamilies (see the Pfam database; El-Gebali et al. 2019). One subfamily (PF00061) consists of ~ 4000 Pfam entries that are mostly (88%) from Metazoan species, and includes LCN2, whereas the other subfamily (PF08212) consists of ~ 3000 entries, mostly from (67%) Bacteria. Lipocalins are predominantly (92%) single domain proteins and multiple homologues are found in vertebrates (e.g. there are 37 lipocalins identified in the human genome; Du et al. 2015).

Since lipocalins are found throughout most of the living kingdom, it may not be surprising to find that they are present in egg white. Extracellular fatty-acid-binding protein (Ex-FABP) was the first lipocalin-like protein identified in egg white and was discovered by a proteomic analysis of hen egg white using 2-dimensional gel electrophoresis (2-DE) followed by liquid chromatography-mass spectroscopy (LC-MS/MS) (Desert et al. 2001). Further work by Guérin-Dubiard et al. (2006) using 2-DE, LC-MS/MS and MALDI-TOF identified a total of 16 proteins in hen egg white, including Ex-FABP as well as two other lipocalin-like proteins: chondrogenesis-associated lipocalin (Cal-γ or prostaglandin D synthase) and α-1-ovoglycoprotein. The presence of the three lipocalin-like proteins in egg white was later confirmed by further proteomic analyses involving 1-DE with LC–MS/MS, 2-DE combined with protein-enrichment (peptide ligand libraries) technology, and a dual-pressure linear-ion-trap Orbitrap instrument (LTQ Orbitrap Velos) (Mann 2007; D’Ambrosio et al. 2008; Mann and Mann 2011).

Although the concentrations of the three lipocalin-like proteins have not been reported, in the Guérin-Dubiard et al. (2006) study the intensity of Cal-γ and Ex-FABP 2-DE spots were weak indicating a very low concentration. In other work (Mann 2007; Mann and Mann 2011), the exponentially-modified-protein abundance index (emPAI) was used to provide an estimate of the absolute abundance of each egg-white protein, which indicated that the lipocalin-like proteins belong to the ‘minor proteins’ set (such as avidin, cystatin, apolipoprotein D, HEP21, Defensin-11) rather than the ‘major proteins’ set (such as ovalbumin, ovotransferrin, lysozyme, ovomucoid and ovoinhibitor). However, as α-1-ovoglycoprotein is glycosylated its detection might be obscured (Mann 2007). In summary, although several studies have shown that three lipocalin-like are present in egg white, their exact concentrations remain unclear. Therefore, their biological significance in egg white remains to be established.

### Sequestration of bacterial siderophores by the lipocalin-like Ex-FABP protein found in egg white

#### Ex-FABP was first discovered as a fatty-acid-binding protein with a role in hen-embryo development

Cancedda et al. (1988) were the first to report and identify Ex-FABP (Ch21) as a protein expressed and secreted by in vitro differentiating hen chondrocytes at a late stage of development. Ex-FABP was later shown to be a 21 kDa protein in cartilage (Cancedda et al. 1988), muscle tissue (Gentili et al. 1998) and granulocytes (Dozin et al. 1992) of chicken embryos. This protein was classified as a member of the superfamily of lipocalins and thus was considered to have a likely role in the transport of small hydrophobic molecules (Cancedda et al. 1990). The protein was renamed (from CH21) ‘extracellular fatty acid-binding protein’ because of its ability to selectively bind and transport fatty acids (i.e. oleic, linoleic, and arachidonic acid) in extracellular fluids and serum (Cancedda et al. 1996). It was shown to be expressed during muscle-fibre formation (Gentili et al. 1998) and later shown to have involvement in endochondral-bone formation (Cermelli et al. 2000; Gentili et al. 2005). Transfection of proliferating chondrocytes and myoblasts with an expression vector expressing antisense Ex-FABP cDNA led to a decreased cell viability. Therefore, Ex-FABP seems to play a part in cell differentiation and cell survival (Di Marco et al. 2003; Gentili et al. 2005). It was more recently shown that Ex-FABP binds the C16 and C18 isoforms of lysophosphatidic acid (LPA, 1- or 2-acyl-*sn*-glycerol-3-phosphate) (Correnti et al. 2011). LPAs are phospholipids mediating differentiation, inflammation, immune function, oxidative stress, cell migration, smooth muscle contraction, apoptosis and development (Zhao and Natarajan 2014). It is likely that the functions of Ex-FABP reported above depend on its role in sensing or transporting phospholipids (Sia et al. 2013).

#### Ex-FABP also binds siderophores and inhibits bacterial growth

**Fig. 3:**
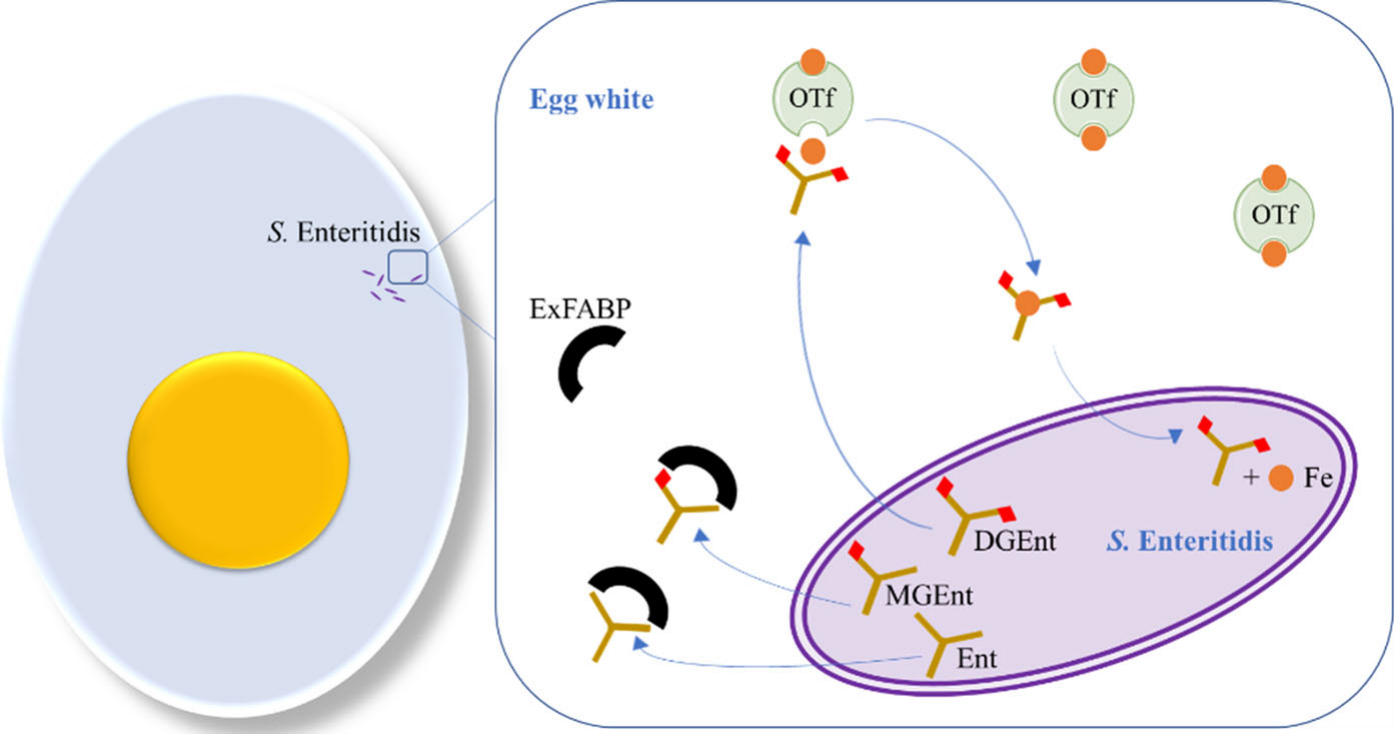
Potential interactions between *S*E siderophores and Ex-FABP. In the egg white, enterobactin (Ent) and MGEnt might be sequestrated by Ex-FABP (represented by black semi-circles), while DGEnt would remain free to chelate iron from ovotransferrin (OTf indicated as green) and thus provide iron to *S*E (glucosyl groups are shown as red diamonds). After import into the cytoplasm, Fe^3+^ (drawn as orange circles) is finally released into the cytoplasm

More recent reports indicate that Ex-FABP functions in pathogen defence through an ability to bind siderophores, in a manner analogous to that of LCN2 (Correnti et al. 2011; Garénaux et al. 2013). This suggests that Ex-FABP may have two distinct purposes, one in fatty acid/LPA binding and another as a siderophore-binding factor. Work of Correnti et al. (2011) shows that Ex-FABP sequesters ferric-enterobactin, as well as its mono-glucosylated (Fe-MGEnt) form with a K_d_ of 0.22 and 0.07 nM, respectively; but not its di-glucosylated form (Fe-DGEnt; K_d_ > 600 nM). Furthermore, Ex-FABP at 5 µM caused growth inhibition of both *E. coli* and *Bacillus subtilis* under iron-limited in vitro conditions. Growth was restored by supplementing the cultures with stoichiometric amounts of FeCl_3_ (Correnti et al. 2011). Thus, Ex-FABP might act to reduce bacterial growth in egg white by enhancing iron restriction (Fig. 3). Ex-FABP did not inhibit *Pseudomonas aeruginosa* growth under iron limitation, which correlates with the observation that Ex-FABP does not bind the corresponding siderophores. Indeed, both enterobactin and bacillibactin produced by *E. coli* and *B. subtilis* (respectively) were found to be sequestrated by Ex-FABP (K_d_ of 0.5 and 30 nM, respectively), while pyochelin and pyoverdine produced by *P. aeruginosa* were not (Correnti et al. 2011). These findings are also in accordance with those from Garénaux et al. (2013) showing that *E. coli* K-12 is subject to a 10^5^-fold growth reduction when exposed to 2.5 µM Ex-FABP or LCN2. However, when transformed with a plasmid harbouring the *iroBCDEN* cluster, no growth defect was observed by 2.5 µM Ex-FABP or LCN2. Exposure of six poultry APEC isolates to 2.5 µM Ex-FABP or LCN2 inhibited the growth of strains producing enterobactin as sole siderophore, but not those producing additional siderophores (salmochelin, aerobactin and/or yersinabactin) (Garénaux et al. 2013). Therefore, it can be concluded that Ex-FABP is an avian siderocalin-type lipocalin with a function similar to that of LCN2.

**Fig. 4:**
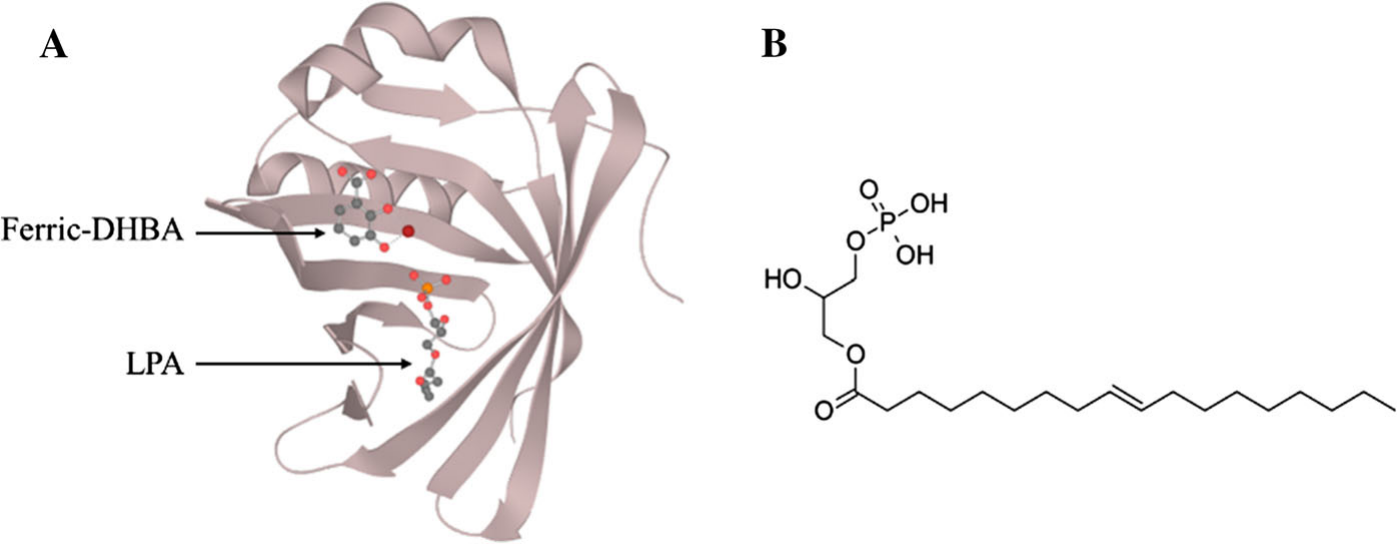
**a** X-ray diffraction (1.8 Å resolution) of Ex-FABP; two ligands are represented, ferric-DHB in the upper cavity and LPA  in the lower cavity. Structure extracted from Protein Data Bank (PMID: 22153502; Correnti et al. 2011). **b** Structure of a C18 lysophosphatidic acid (LPA)

The pleiotropic function (i.e. siderophore and LPA binding) of Ex-FABP might be explained by the large binding site of the molecule (Sia et al. 2013). Ex-FABP has a three-dimensional fold common to that of lipocalin family proteins but has an extra α-helix (residues 22–30) and short helical element (residues 139–141). This results in an extended calyx that encompasses upper and lower cavities (Fig. 4). The upper cavity comprises a siderophore-binding site with three catechol-binding pockets involving basic residues (K82, R101 and R112) key to ligand binding (Correnti et al. 2011). K82 forms hydrogen bonds with the 3-OH of the catechol groups, while R101 and R112 provide significant electrostatic contributions to ligand-binding. The lower cavity acts as a hydrophobic binding site that can bind C16 and C18 LPA. Modelling of the complex shows that the side-chains Y50, K82, R112 and Y114 of Ex-FABP make hydrogen bonds with LPA (Correnti et al. 2011).

### The other lipocalins of egg white

#### Phylogenic relationship

**Fig. 5:**
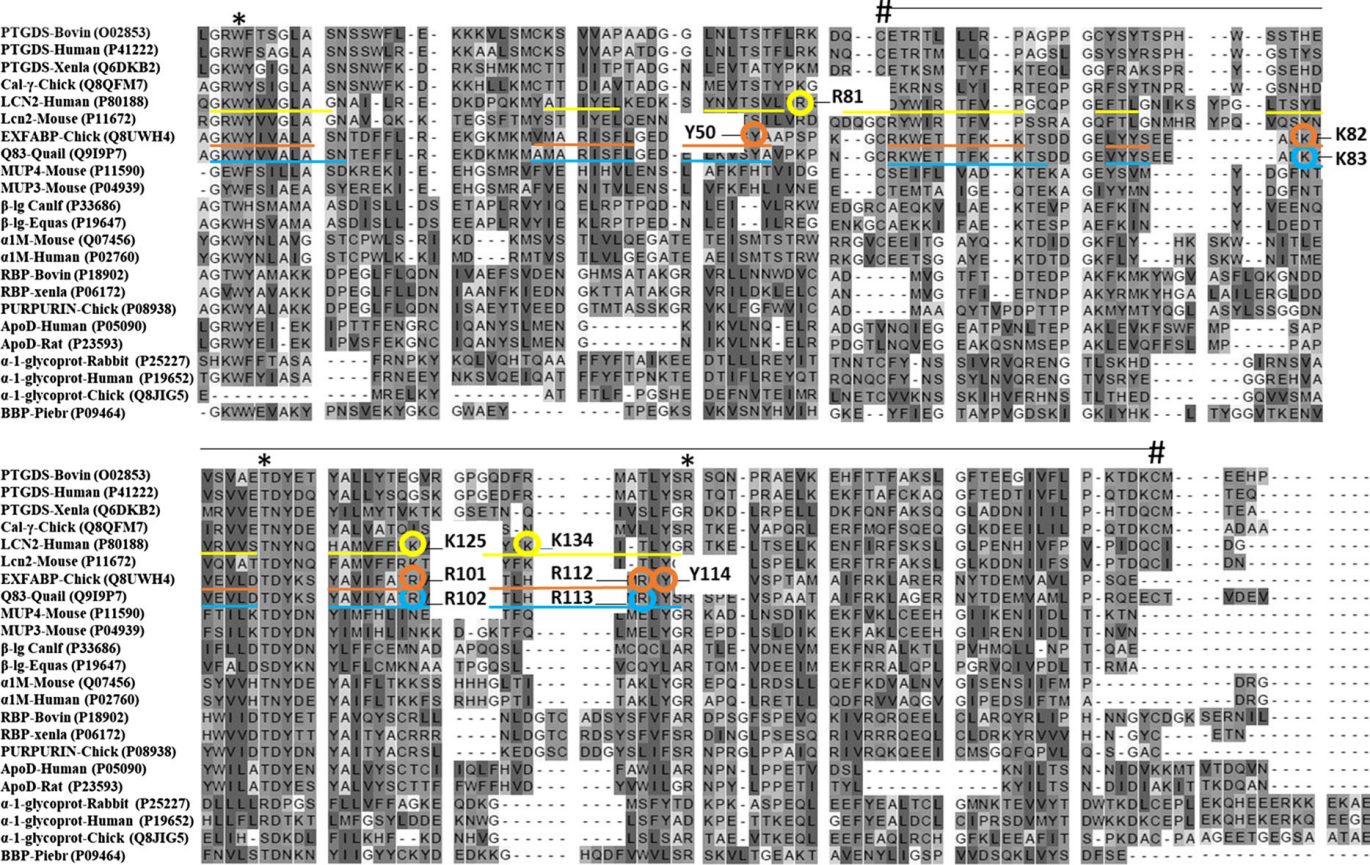
Multiple-sequence alignment (Genomic Workbench) of lipocalin-like proteins found in egg white with their closest homologues extracted from the Pfam database (El-Gebali et al. 2019) and NCBI (2019). The Uniprot accession number of each protein is in brackets. Three motifs are shown (*) centred on the conserved tryptophan, threonine and arginine residues. These residues are preserved in lipocalins and seal the bottom end of the barrel, along with the 3_10_-helix (Bao et al. 2015). Two cysteine residues (#) playing a role in disulphide bridging are also conserved. Residues (putatively) involved in siderophore binding are circled in yellow for LCN2: R81, K125, K134 (Goetz et al. 2002) and blue for Q83: K83, R102, R113 (Coudevylle et al. 2010). For Ex-FABP, residues involved in siderophore (K82, R101, R112) and LPA (Y50, K82, R112, Y114) binding are circled in orange. The β-strands of the eight-stranded β-barrel of the LCN2, Q83 and Ex-FABP are underlined in yellow, orange and blue, respectively

**Fig. 6:**
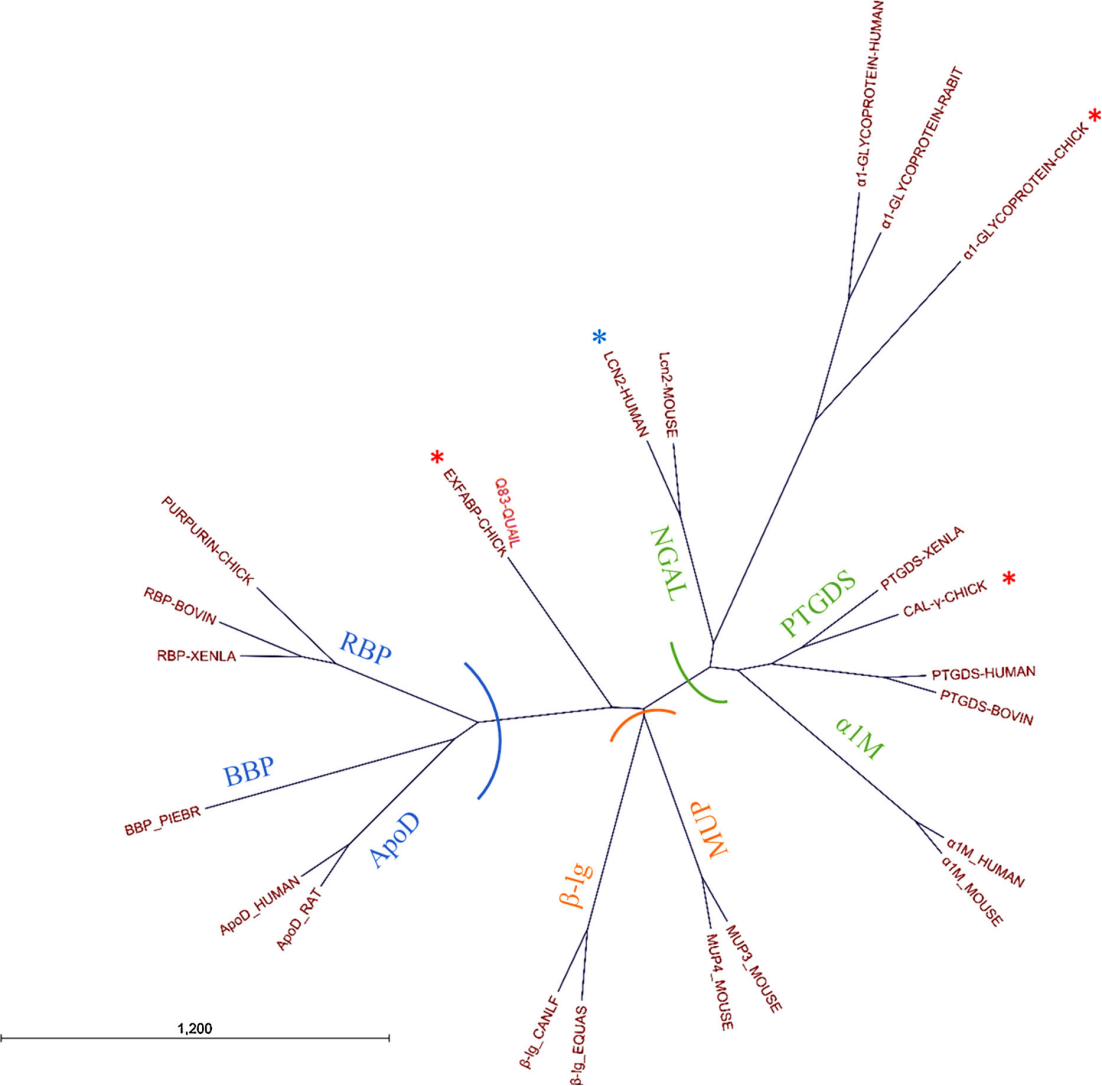
Neighbour-joining tree of the lipocalin-like proteins found in egg white and their closest homologues. The alignment of lipocalin-like proteins (Fig. 5) extracted from the Pfam family PF00061 and NCBI (2019) was used to build the phylogeny tree with CLC Genomics Workbench (protein distance was estimated from the Jukes-Cantor model). Three lobes are identified with coloured half-circles (green for NGAL, PTGDS and α1M; orange for β-lg and MUP; blue for RBP, BBP and ApoD). Red stars show the lipocalin homologues found in hen egg white. The blue star indicates human lipocalin-2

A multiple-sequence alignment and phylogenetic analysis of the three lipocalin-like proteins of egg white is presented in Figs. 5 and 6. The tree can be organised into three lobes, as described by Flower et al. (2000): proteins in the green lobe include prostaglandin D synthase (PTGDS), neutrophil lipocalin and α-1-microglobulin (α1 M); the blue lobe includes bilin-binding protein (BBP), retinol-binding protein (RBP) and apolipoprotein D (apoD); and the orange lobe is formed of major-urinary protein (MUP) and β-lactoglobulin (β-lg). This phylogenetic analysis indicates that both Cal-**γ** and α-1-ovoglycoprotein have a human orthologue. This is in accordance with a comparative analysis of the chicken genome that showed that 60% of chicken protein-coding genes have a single human orthologue (Consortium ICGS 2004). This tree also indicates that LCN2 from *H. sapiens* is more closely related to Cal-γ than to the other two lipocalin-like proteins found in chicken egg white, and that α-1-ovoglycoprotein could be considered as an outlier among the lipocalin family. Yet, despite their limited sequence identities (26%; NCBI 2019), hen Ex-FABP and human LCN2 have similar ligand-binding affinities (Correnti et al. 2011). According to Fig. 6, there is a close homologue of known function to Ex-FABP found in quail: the Q83 lipocalin (88% identity; NCBI 2019). Q83 was originally identified based on its overexpression in quail embryo fibroblasts transformed with the v-myc oncogene. Q83 sequesters enterobactin with a mode of binding equivalent to that of LCN2 (Coudevylle et al. 2010). This resembles Ex-FABP’s function, as described above, in siderophore inhibition, and is consistent with its presence in egg white. Surprisingly, there is no close homologue of Ex-FABP in man, nor of LCN2 in chicken: the closest human homologue of Ex-FABP is lipocalin 15 and the closest chicken homologue of LCN2 is Cal-γ (28 and 30% amino-acid sequence identity, respectively; NCBI 2019). This suggests that the siderophore-binding activities of these two proteins have evolved independently, in related proteins, in order to fulfil similar functional requirements in innate immunity.

#### Alpha-1-ovoglycoprotein and Cal-γ: their potential functions in egg white

Alpha-1-ovoglycoprotein (or ‘orosomucoid’) shares a closely-related common ancestor with other α1-acid glycoproteins found in various animals (Fig. 6). Despite its induction as an acute-phase protein, and its role in cellular inflammation and transport of drugs in human serum, its biological purpose is unclear (Huang and Ung 2013). In man, this protein is found in the serum where it is heavily glycosylated and highly acidic due to the presence of sialic acid. Human α1-acid-glycoprotein (α1-AGP) is a highly glycosylated protein (approximatively 45%) of 43 kDa with a pI of 2.7 (Schmid 1975). There are at least two genes encoding α1-AGP identified, thus it has been suggested that the protein found in plasma is a mixture of the products of these two distinct genes (Dente et al. 1985). The normal plasma concentration in man is between 0.7 and 1.0 g/L. However, as an acute-phase protein, its concentration can increase to 3 g/L under inflammatory conditions (Kremer et al. 1988). It is known to bind lipopolysaccharide and can stimulate the activation of inflammatory cell lines (Boutten et al. 1992). Interestingly, α1-AGP has a protective effect in a mouse meningococcal shock model, suggesting a potential antibacterial role (Moore et al. 1997). In egg white, the α-1-ovoglycoprotein has an average molecular weight of 30 kDa, an isoelectric point of 4.37–4.51 and a sugar content of about 25% (Matsunaga et al. 2004). While little is known about its function, this ovoglycoprotein is often used for its chiral properties to separate drug enantiomers (Sadakane et al. 2002; Haginaka and Takehira 1997). However, its biochemical, functional and biological properties in egg white remain unknown.

The phylogenetic analysis (Fig. 6) indicates that the closest homologue of Cal-γ is lipocalin-like prostaglandin synthase (PTGDS). In mammals, PTGDS is secreted into various body fluids. This protein catalyses the isomerization of prostaglandin H2 to prostaglandin D2, and was reported to bind a variety of lipophilic molecules such as biliverdin, bilirubin and retinoic acid. In humans, this protein is likely to be involved in both maturation and maintenance of the central-nervous system and male reproductive system (Saito et al. 2002; Urade and Hayaishi 2000). Two isoforms, of 22 kDa, can be separated by 2D-electrophoresis of egg white, thanks to their different isoelectric points (pI of 5.6 and 6.0) (Guérin-Dubiard et al. 2006). Pagano et al. (2003) have shown that Cal-γ expression correlates with endochondral bone formation and the inflammatory response. As for Ex-FABP, Cal-γ mRNA is increasingly synthesized during chondrocyte differentiation both in vivo and in vitro. Although Ex-FABP and Cal-γ may both play a part in bone formation and the inflammatory response, any possible role for Cal-γ in siderophore sequestration remains to be explored.

## Conclusion

The antibacterial iron-restriction activity of egg white, as mediated by oTf, is well established and it is now apparent that the egg-white lipocalin, Ex-FABP, can inhibit bacterial growth via an enhanced iron-restriction effect that is mediated by its siderophore-binding capacity. However, the siderophore-sequestering activity of Ex-FABP has neither been studied in egg white, nor with appropriate *S*E or hen infection models. Furthermore, how and in what quantity this protein is incorporated into egg white remains unknown. As yet, it is unclear whether the other lipocalins of egg white (Cal-γ and α-1-glycoprotein) might also sequester siderophores. Although many egg-white proteins have been shown to be components of the arsenal of defence factors within egg white, the contribution (if any) of the three lipocalins as new egg-white defence factors remains an open question, although this now appears highly likely for Ex-FABP. As matters stand, it is unclear whether the capacity of salmochelin to assists *S*E virulence in mammalian models can be extended to include support of *S*E survival in egg white. Thus, there remains much scope for further understanding of the role of lipocalin proteins in the defence of egg white against bacteria.
